# Periodic Email Prompts to Re-Use an Internet-Delivered Computer-Tailored Lifestyle Program: Influence of Prompt Content and Timing

**DOI:** 10.2196/jmir.2151

**Published:** 2013-01-31

**Authors:** Francine Schneider, Hein de Vries, Math Candel, Angelique van de Kar, Liesbeth van Osch

**Affiliations:** ^1^CAPHRIDepartment of Health PromotionMaastricht UniversityMaastrichtNetherlands; ^2^CAPHRIDepartment of Methodology and StatisticsMaastricht UniversityMaastrichtNetherlands; ^3^Veiligheidsregio Limburg-NoordNijmegenNetherlands

**Keywords:** Computer tailoring, Internet-delivered intervention, intervention adherence, periodic email prompts, prompt content, prompt timing

## Abstract

**Background:**

Adherence to Internet-delivered lifestyle interventions using multiple tailoring is suboptimal. Therefore, it is essential to invest in proactive strategies, such as periodic email prompts, to boost re-use of the intervention.

**Objective:**

This study investigated the influence of content and timing of a single email prompt on re-use of an Internet-delivered computer-tailored (CT) lifestyle program.

**Methods:**

A sample of municipality employees was invited to participate in the program. All participants who decided to use the program received an email prompting them to revisit the program. A 2×3 (content × timing) design was used to test manipulations of prompt content and timing. Depending on the study group participants were randomly assigned to, they received either a prompt containing standard content (an invitation to revisit the program), or standard content plus a preview of new content placed on the program website. Participants received this prompt after 2, 4, or 6 weeks. In addition to these 6 experimental conditions, a control condition was included consisting of participants who did not receive an additional email prompt. Clicks on the uniform resource locator (URL) provided in the prompt and log-ins to the CT program were objectively monitored. Logistic regression analyses were conducted to determine whether prompt content and/or prompt timing predicted clicking on the URL and logging in to the CT program.

**Results:**

Of all program users (N=240), 206 participants received a subsequent email prompting them to revisit the program. A total of 53 participants (25.7%) who received a prompt reacted to this prompt by clicking on the URL, and 25 participants (12.1%) actually logged in to the program. There was a main effect of prompt timing; participants receiving an email prompt 2 weeks after their first visit clicked on the URL significantly more often compared with participants that received the prompt after 4 weeks (odds ratio [OR] 3.069, 95% CI 1.392-6.765, *P*=.005) and after 6 weeks (OR 4.471, 95% CI 1.909-10.471, *P*=.001). Furthermore, participants who received an email prompt 2 weeks after their first visit logged in to the program significantly more often compared to participants receiving the prompt after 6 weeks (OR 16.356, 95% CI 2.071-129.196, *P*=.008). A trend was observed with regard to prompt content. Participants receiving a prompt with additional content were more likely to log in to the program compared to participants who received a standard prompt. However, this result was not statistically significant (OR 2.286, 95% CI 0.892-5.856, *P*=.09).

**Conclusions:**

The key findings suggest that boosting revisits to a CT program benefits most from relatively short prompt timing. Furthermore, a preview of new website content may be added to a standard prompt to further increase its effectiveness in persuading people to log in to the program.

## Introduction

Internet-delivered lifestyle interventions applying computer-tailoring techniques [1-3] have reported positive effects for multiple health behaviors, such as physical activity [4,5], fruit and vegetable intake [6,7], smoking cessation [8-11], and alcohol consumption [12,13]. Furthermore, providing computer-tailored (CT) advice on multiple occasions (multiple tailoring) has proven to significantly add to their impact [7,14]. However, despite promising prospects, actual exposure to these interventions remains limited [15]. Exposure not only refers to the level of first-time use of the intervention, but also to the quality and quantity of intervention use [16,17]. A small proportion of the potential target population actually accesses the intervention [16,18,19], and the level of adherence to these interventions is even lower [20], making attrition a common and urgent problem in Internet-based trials [15,21]. Non-usage attrition, in which participants lose interest in the intervention and refrain from continued use, is hindering actual impact on public health [17,21-25].

Research on non-usage attrition has demonstrated an initial rapid decline in program use over the first few weeks [15,21]. During these weeks, participants lose interest in the program or realize that it does not meet their wishes or expectations. However, achieving health behavior change is a complex and lengthy process requiring continuous guidance to maximize intervention effects [22,26]. Therefore, repeated use of interventions using multiple tailoring must be stimulated to allow processing of the entire intervention content and engagement in its effective components [21,27-29]. Furthermore, re-use is important because it offers visitors the opportunity to self-monitor their level of behavior change and receive additional personalized advice regarding strategies to increase or maintain their current level of behavior change [30]. It is essential to minimize non-usage attrition at an early stage by investing in strategies that boost revisits to Internet-delivered interventions using multiple tailoring.

A substantial amount of Internet-delivered interventions use reactive strategies to achieve re-use, implying that a passive approach is used in which users themselves must undertake action to repeatedly benefit from the intervention content [31]. However, because preventing non-usage attrition is a very strenuous process [21,25,32], efforts should be put into attaining loyalty to the intervention directly after initiation by utilizing more proactive strategies [17]. The use of periodic email prompts has been proposed as an effective proactive strategy to boost re-use of interventions [33]. Although the number of interventions employing proactive strategies is increasing, most studies merely explored the efficacy of the whole intervention, including prompting, instead of focusing on the added value of periodic prompting as a strategy to boost re-use. Furthermore, those studies indicating that sending periodic email prompts significantly increased program re-use [17,33] recommended further examination and refinement to maximize their potential [17].

More specific evidence on the positive effects of the use of email to promote direct action stems from the field of e-marketing [34,35]. Within this field, the use of email advertisements is rapidly growing because of the increase in email users and their potential to reach large numbers of people at relatively low cost and effort. In addition, in the field of health promotion, emails have been used to prevent attrition in Web-based trials. Several studies have indicated that sending email reminders is an effective strategy to increase response rates in online data collection [36-38]. Furthermore, within one of our previous studies that examined the effect of using periodic email prompts [17], a positive, however modest, effect on re-use of the program was found. Within that study, an email prompt was sent 3 months after a first visit to the intervention. This modest effect might imply that a 3-month period is too long, causing people to forget about the program and their participation, which is in line with previous studies pointing out an elevated non-usage attrition level at the beginning of the intervention period [15,21]. Therefore, we recommended future research to focus on testing strategies to optimize the effect of email prompts [17]. It is imperative to investigate the effect of using shorter prompting intervals and to determine the optimal interval at which prompts should be sent. In contrast, this modest effect of a periodic email prompt might also imply that prompt content is suboptimal. Since people tend to disengage from the intervention relatively shortly after intervention initiation because of a reduced levels of interest [21], involvement in the intervention content and subsequent sessions is likely to decrease. According to the elaboration likelihood model (ELM), people with a low level of involvement are less likely to process arguments used [39]. As a result, argument-based persuasion techniques used in an email prompt persuading participants to re-use the intervention might be insufficiently processed. To increase persuasiveness of the email prompts for people with a low level of involvement in the issue, the current study tested the effect of adding a peripheral cue to a standard email prompt [39]. This peripheral cue consisted of the addition of a preview of new website content. This preview served as a teaser to increase curiosity for the remaining website content [40]. Instead of basing a decision to re-use the program on the argument posed in the email, participants might simply react out of sheer curiosity [39]. It was hypothesized that a prompt with additional content was more effective in persuading people to revisit the CT lifestyle program compared to a standard prompt.

This study aimed to assess the added value of periodic email prompts to boost revisits to an Internet-delivered CT lifestyle program. We aimed to answer 2 questions: (1) which prompt timing interval is most effective in boosting re-use of the Internet-delivered CT lifestyle program and (2) which prompt content is most effective in boosting re-use of the program? We addressed these questions among participants in an Internet-delivered CT intervention aimed at multiple health behaviors: increasing physical activity, increasing fruit and vegetable intake, smoking cessation, and decreasing alcohol consumption.

## Methods

### Procedure and Participants

This study was conducted in close collaboration with the Regional Public Health Service (RPHS) in the northern part of the Dutch region, Limburg. All people employed by the municipalities in this region were invited by the RPHS to participate in an Internet-delivered CT lifestyle program developed by our research group [41,42]. Invitations for this program were placed on the intranet of all municipalities and were also directly emailed to employees. The invitation contained a uniform resource locator (URL) that directed interested participants directly to the program. The program provided employees the opportunity to receive free-of-charge CT feedback about their current health behavior (physical activity, fruit and vegetable intake, alcohol consumption, and smoking) and assistance in changing these health behaviors.

Participants who logged in to the program to obtain CT advice were sent an email prompting them to re-use the program. Re-use was encouraged to allow participants to monitor their behavior change and to obtain iterative CT advice aimed at behavior change and relapse prevention. Furthermore, participants were offered an opportunity to obtain CT advice regarding an additional health behavior. Finally, re-use was stimulated to keep participants informed about new content added to the program website.

### Design

A 2×3 design was used to test the effect of 2 factors: prompt content (standard and standard+) and prompt timing (2, 4, and 6 weeks). The standard prompt (SP) contained a message reminding people about their previous visit to the program and invited them to re-use the program to monitor their progress and obtain additional feedback. The second version of the prompt (SP+) contained standard content complemented with a message alerting people to new content added to the intervention website. Participants received an email prompt after 2, 4, or 6 weeks. In addition to these 6 experimental conditions, a control condition was included, no prompt (NP). People allocated to the control condition did not receive an additional prompt and were only encouraged at baseline to re-use the program. To participate in the program, participants had to register by using a personal log-in code and password. Immediately after registration for the program, participants were randomly allocated to 1 of the 7 study conditions. Randomization occurred at the respondent level by means of a computer software randomization device. Data for the present study were collected from March to July 2011.

### Email Prompt

People who were allocated to the experimental conditions of the study received an email prompting them to re-use the program. Periodic prompts should be distinguished from reminders, which are also often used in Internet-delivered interventions. Sending reminders is a technique that is used to increase response rates in online data collection by proactively stimulating participation among nonresponders and is used to prevent drop-out attrition [21,43]. Periodic prompts, on the other hand, are used to boost re-use of the intervention content by approaching all participants and are used to prevent non-usage attrition [21]. All email prompts used in the present study contained standard content. This standard email opened with a personalized greeting and reminded people about their first visit to the program. Subsequently, people were invited to re-use the program to obtain information about their current health status and to monitor their progress. Participants were also given the opportunity to receive additional iterative health advice on the health behavior(s) selected at baseline or on a new behavior. Finally, to facilitate logging in to the program, the email also contained details about their personal log-in information (username and password). The email concluded with greetings from the research team and contact information. Half of all people in the experimental conditions received an email that also contained additional content (SP+). This additional content consisted of a preview of new information that was placed on the program website since they last visited it. This information referred to nutrition and provided examples of healthy food alternatives that were available for that current season (eg, spring/summer).

### Internet-Delivered Computer-Tailored Lifestyle Program

The CT program integrated established CT programs tested and proven to be effective in randomized controlled trials for increasing smoking cessation, promoting the intake of fruit and vegetables, increasing the level of physical activity, and reducing the consumption of alcohol [44-48]. The program used a dual approach to guide people toward behavior change. First, awareness of participants’ current health behavior status was increased by comparing their status to the Dutch public health guidelines set for these health behaviors, such as being moderately physically active for 30 minutes at least 5 days a week, eating 2 pieces of fruit per day, eating 200 grams of vegetables per day, not drinking more than 1 (women) or 2 (men) glasses of alcohol a day, and not smoking. Second, assistance was provided in changing participants’ health behavior by using CT modules available per behavior. The modules used a fixed, gradual approach consisting of 4 steps, guiding people toward behavior change based on the Integrated Model for exploring motivational and behavioral change (I-Change Model) [49]. Focus was on pros and cons of the desired behavior (step 1), the role of significant persons in the direct environment (step 2), preparatory plans assisting people to start changing their behavior (step 3), and coping plans to help them overcome difficult situations and prevent relapse (step 4). Within the modules, all health advice was adapted to the individual’s characteristics by considering demographic, behavioral, and cognitive characteristics [50-52]. The Internet-delivered CT lifestyle program is described in more detail elsewhere [41,42].

The program was embedded in a website designed for the current study. This website entailed general information considering a healthy lifestyle and the selected health behaviors. Furthermore, the website provided specific information regarding the project, contained a direct link to the CT program, and provided background information regarding the study and the research team. During the study, new information (eg, advice, supporting messages, recipes, and facts) was added to the website.

### Measures

Participants in the experimental conditions (SP and SP+) received an email prompting them to re-use the intervention. Participants had to take 2 steps to re-use the program in order to self-monitor their level of behavior change and obtain additional CT advice. The first was clicking on the URL provided in the email prompt. Clicking on the URL was tracked by referral ID codes that were integrated in the URL. The second step was logging in to the CT program after arriving on the program website. Log-ins were objectively monitored during a 2- week period and log-in dates were compared to baseline dates to determine revisits.

To describe characteristics of program visitors, age, gender, and educational level of participants was assessed (1/low: no education or lower vocational school; 2/medium: secondary vocational school or high school; 3/high: higher professional education or university). Health behavior status consisted of information regarding the 5 key behaviors. Physical activity was measured by the Short Questionnaire to Assess Health-enhancing physical activity (SQUASH) [53] and guideline adherence was calculated following procedures used by Ainsworth et al (2000) [54]. Fruit consumption was measured by using a 4-item Food Frequency Questionnaire (FFQ) assessing weekly amount of fruit and fruit juice intake [55], whereas vegetable consumption was measured using a 4-item FFQ assessing the weekly amount of consumed boiled or baked vegetables as well as salad or raw vegetables [55]. The consumption of alcohol was measured by the Dutch Quantity-Frequency-Variability (QFV) questionnaire [56]. Finally, smoking status was assessed by asking participants whether they smoked, what they smoked (cigarettes, cigars, pipe tobacco), and how much they smoked per day (cigarettes) and per week (cigars/packets pipe tobacco) [57]. For each health behavior, a new variable was created to indicate whether participants met the Dutch guidelines provided for these behaviors (0=no; 1=yes).

### Statistical Analysis

General descriptive statistics were calculated to describe characteristics of program visitors, as well as main findings concerning adherence to the public health guidelines. Baseline differences between the intervention groups were calculated using the Chi-square test for dichotomous and categorical variables and 1-way analysis of variance (ANOVA) for continuous variables. Finally, logistic regression analyses were conducted. There were 2 dependent variables: whether participants clicked on the URL (0=no; 1=yes) and whether participants logged in to the CT program (0=no; 1=yes). Prompt content (dummy coded, SP=0; SP+=1) and prompt timing (dummy coded with 2 weeks as the reference category and 4 weeks as the reference category) and the interactions between these variables were used as predictors in the initial model of each dependent variable. Furthermore, because women and people with a higher educational level and age are more likely to use Internet-delivered lifestyle interventions [17,58], age, gender, and educational level were included in the models as possible covariates. An alpha of .05 was used to indicate statistical significance. All statistical analyses were done with the program SPSS 17.0 (SPSS Inc, Chicago, IL, USA).

## Results

In total, 240 participants visited the program, of which 73.3% (176/240) were female. Participants were randomly allocated to each of the 7 study conditions and a randomization check revealed that females were equally distributed (χ^2^_6_= 9.1, *P*=.17). Furthermore, there were no significant differences between the groups regarding educational level (χ^2^_12_= 9.3, *P*=.68) and age (*F*_6,233_= 0.464, *P*=.84). Overall visitors had a mean age of 50 years (SD 14.99) and most were medium (100/240, 41.7%) to highly educated (111/240, 46.3%). Regarding the 5 health behaviors included in the program, 12.9% (31/240) did not comply with the Dutch guidelines of at least 30 minutes of moderately intensive physical activity on at least 5 days of the week. With regard to fruit and vegetable intake, 50.8% (122/240) and 60.0% (144/240) did not adhere to the Dutch guidelines of at least 2 pieces of fruit and at least 200 gram of vegetables each day, respectively. Approximately 1 out of 10 participants indicated that they smoked (10.0%, 24/240), and 22.1% (53/240) did not comply with the Dutch guidelines for alcohol intake.

A total of 206 participants received an email prompting them to re-use the program. Of this sample, 53 participants (25.7%) reacted to this email by clicking on the URL (step 1), whereas 25 participants (12.1%) actually logged in to the program (step 2). All results concerning clicking on the URL and logging in to the program are described per study group in Table 1.

**Table 1:** Participants who clicked on the URL (step 1) and logged in to the program (step 2) per study condition (N=240).

Dependent variable	Condition, n (%)						
	Standard content			Standard+ content			No prompt
	2 weeks n=34	4 weeks n=34	6 weeks n=35	2 weeks n=36	4 weeks n=35	6 weeks n=32	n=34
Click on the URL	14 (41.2)	6 (17.6)	4 (11.4)	16 (44.4)	7 (20.0)	6 (18.8)	—
Log in to the program	6 (17.6)	1 (2.9)	1 (2.9)	10 (27.8)	7 (20.0)	0 (0.0)	2 (5.9)

### Step 1: Clicking on the URL

**Figure 1:**
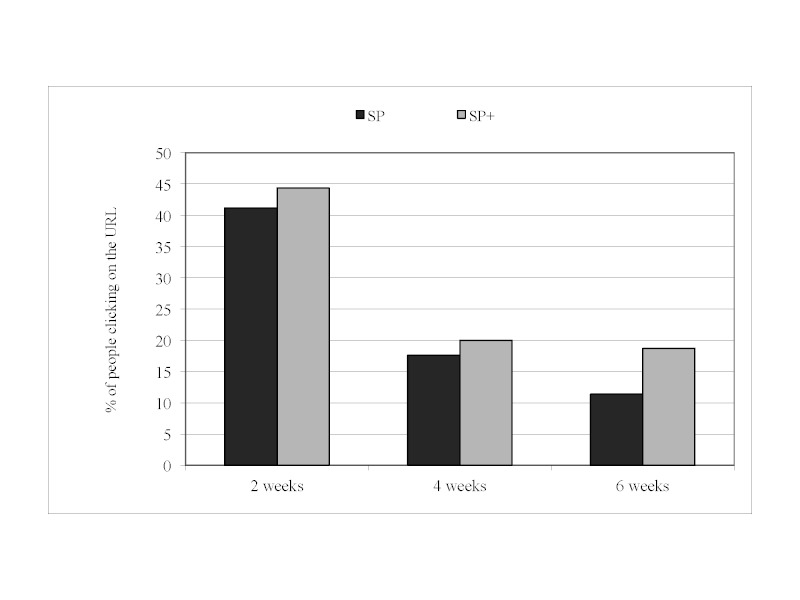
Percentage of participants given standard prompts (SP) and standard+ prompts (SP+) who clicked on the URL at different levels of prompt timing.

There was no significant interaction between prompt content and prompt timing (Figure 1) with regard to clicking on the URL. Therefore, interaction terms were excluded from the remaining models and only main effects are reported.

Analyses of main effects indicated that there was a significant effect of prompt timing (χ^2^_2_ = 15.2, *P* = <.001). Participants who received an email prompt 2 weeks after their first visit, clicked on the URL significantly more often compared with participants that received the prompt after 4 weeks (odds ratio [OR] 3.069, 95% CI 1.392-6.765, *P*=.005) and after 6 weeks (OR 4.471, 95% CI 1.909-10.471, *P*=.001). There was no significant difference in reaction to the email prompt between participants receiving the prompts after 4 weeks, compared with participants receiving the prompts after 6 weeks. Also, no main effects of prompt content could be detected (see Table 1).

### Logging In to the Program

**Figure 2:**
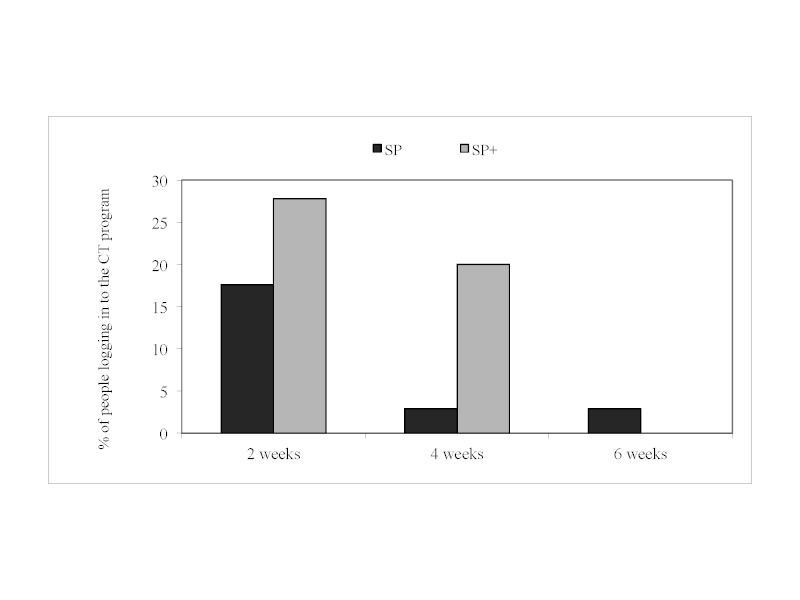
Percentage of participants given standard prompts (SP) and standard+ prompts (SP+) who logged in to the program at different levels of prompt timing.

With regard to logging in to the program, no significant interaction between prompt content and prompt timing was found (Figure 2). Therefore, interaction terms were again excluded from the remaining models and only main effects are reported.

With regard to logging in to the program, analyses of main effects indicated that there was a significant effect of prompt timing (χ^2^_2_ = 16.5, *P*<.001). Participants receiving an email prompt 2 weeks after their first visit, logged in to the program significantly more often compared to participants receiving the prompt after 6 weeks (OR 16.356, 95% CI 2.071-129.196, *P*=.008). There was, however, no significant difference between participants receiving the prompts after 4 weeks compared with participants receiving the prompts after 2 or 6 weeks. With regard to prompt content, a trend was observed. Participants receiving a prompt with additional content were more likely to log in to the program compared to participants who received a prompt with standard content. However, this result was not statistically significant (OR 2.286, 95% CI 0.892-5.856, *P*=.09).

**Table 2:** Effects of prompt content and timing on clicking on the URL (step 1) and logging in to the program (step 2).

Dependent variable	Condition	OR	95% CI	*P*
Clicking on the URL				
	Prompt content (SP versus SP+)^a^	1.278	0.652-2.505	.48
	Prompt timing (2 week vs 4 week)	3.069	1.392-6.765	.005
	Prompt timing (2 week vs 6 week)	4.471	1.909-10.471	.001
	Prompt timing (4 week vs 6 week)	1.457	0.575-3.689	.43
Logging in to the program				
	Prompt content (SP vs SP+)^a^	2.286	0.892-5.856	.09
	Prompt timing (2 week vs 4 week)	2.144	0.822-5.593	.12
	Prompt timing (2 week vs 6 week)	16.356	2.071-129.196	.008
	Prompt timing (4 week vs 6 week)	0.131	0.016-1.096	.06

^a^SP: standard prompt content; SP+: standard prompt content complemented with a preview of new website content.

Finally, all experimental groups were compared with the control condition (NP) that did not receive an additional email prompt to boost revisits to the intervention. Results from this analysis revealed that participants who received an email prompt containing additional content after 2 weeks were significantly more likely to log in to the program (OR 6.059, 95% CI 1.195-30.726, *P*<.001).

## Discussion

This study aimed at maximizing the potential of email prompts by focusing on prompt content and timing. A single email prompt was used in the context of an Internet-delivered CT lifestyle program and was aimed at stimulating re-use to increase the dose of the intervention and prevent non-usage attrition. Firstly, results indicated that sending a prompt 2 weeks after the first visit was more effective compared with using a longer time period. This effect not only referred to clicking on the URL (step 1), but also to the proportion of log-ins to the program (step 2). Secondly, a trend was observed indicating that adding a preview of new website content to a standard prompt increased its effectiveness in persuading people to log in to the program. Finally, sending a prompt with additional content after a 2-week period significantly increased program log-ins compared to using a reactive approach in which no additional prompts were used.

The effectiveness of using a relatively brief interval for sending a prompt may indicate that people tend to forget about the program. Therefore, the interval between engaging in a first visit and receiving an email prompt to boost re-use should be kept relatively short. Since the costs of sending a large number of email messages are relatively low [59], this strategy allows for sending several emails at relatively short intervals. However, when using an email prompt to boost re-use of the program, one must keep the goal of the revisit in mind. Within the current program, the prompt was used to remind people about their previous visit to the program and to persuade them to re-use the program to monitor their progress and obtain additional feedback. Although results from this study suggest that prompt timing should be short, one must allow participants enough time to actually follow the obtained advice and develop strategies to positively change their lifestyle [32].

Results from the current study also show a trend toward the efficacy of adding additional content to a standard prompt. However, this trend was only present for logging in to the program (step 2) and not for clicking on the URL (step 1). A possible explanation for this effect on log-ins might be the repeated exposure to new website content. People who received an email prompt containing a preview of new website content were exposed to this website content twice: within the email prompt and on the program website. According to ease of processing theory [60], repeated exposure to information allows for more fluent processing of the information. This extended degree of exposure might have enhanced the ease of processing the new information added to the website. Because this new information was related to health and emphasized the importance of having a healthy lifestyle, this information could have persuaded people to monitor their current lifestyle behavior and obtain additional advice about how to positively change their behavior.

With regard to clicking on the URL (step 1), significant effects of adding a peripheral cue to a standard prompt failed to occur. People were significantly more likely to respond to emails that were sent at a relatively short interval, irrespective of the email content. A possible explanation for this finding might be that participants consented to participate in the present study after being informed about the possibility of receiving an additional email to boost program use. Within the field of e-marketing, the term *opt-in email* is used when receivers have agreed to the receipt of an email [34,61]. Research has indicated that opt-in emails are largely accepted among receivers and are proven to be more effective compared to spam email (receiver has not agreed to receiving an email) when used to persuade people to undertake action (eg, clicking on a URL to re-use the program) [34,61]. Therefore, knowledge about receiving an email prompt might have increased acceptance of the email among participants and might have resulted in the desired behavior (clicking on the URL) irrespective of the email content.

### Strengths, Limitations, and Recommendations

The current study primarily focused on the effectiveness of using a single email prompt with regard to clicking on the URL and logging in to the program. Combined with results from our previous study [17], important information regarding the overall effectiveness of using a single email prompt as well as the optimal prompt content and timing was obtained. To our knowledge, this is one of the first studies that systematically studied optimal content and timing of a single email prompt used to boost re-use of a multiple CT lifestyle program offered through the Internet. Although these results suggest that sending a single email prompt is an effective strategy to boost revisits to interventions using multiple computer-tailoring, this is only a first step, and the findings should be interpreted keeping several limitations in mind. Firstly, the current study focused solely on sending an email prompt on 1 occasion, namely after 2, 4, or 6 weeks. However, achieving health behavior change is a complex and enduring process that requires continuous guidance [22,26]. Repeated visits to interventions using multiple computer-tailoring are imperative to ensure notification of the entire intervention content and involvement in its effective components [27,28] and must, therefore, be encouraged. Additional research should go one step further and focus on timing intervals and prompt frequency. Prompting for revisits shortly after a first visit indeed stimulated revisits to the program. However, repeatedly sending prompts at a certain interval to boost multiple revisits might have different or even reverse effects because responses to multiple prompts tend to gradually decline over time [62]. Secondly, effectiveness of the use of an email prompt largely depends on the degree to which the content is actually read and processed by receivers. Within the current study, no objective measure of the degree to which emails were read was obtained. Therefore, results on prompt content should be interpreted with caution. Future research should put additional effort in assessing the degree to which email content is actually read and processed by participants. Furthermore, additional research is needed to investigate the effects of using periodic prompts on behavior change and to investigate whether these effects remain present over a sustained period of time. Thirdly, the sample size for the present study was relatively small. As a result, the study might be relatively underpowered to study interaction effects and to indicate significant main effects. Furthermore, regression analyses resulted in several substantial confidence intervals. Although these results should be interpreted with caution, they still provide valuable information regarding the effectiveness of timing and content of a single email prompt as a strategy to boost program revisits. Fourthly, a related limitation is the representativeness of the study sample. This sample does not provide a good cross-section of the general Dutch population [63] because participants were mainly female, middle-aged, and medium to highly educated. This may limit possibilities for generalizing our results to the general population. However, previous studies repeatedly indicated that Internet-delivered CT programs tend to predominantly reach women and people who are older and higher educated [17,58,64-67]. As a consequence, the current sample corresponds to the subgroup of people known to be reached by Internet-delivered CT programs, although it does not represent the general population. Therefore, the obtained results are valuable in the context of CT lifestyle programs that are offered through the Internet.

### Conclusions

We found that using relatively short prompt timing (2 weeks) resulted in more positive effects compared to using a longer time period. This effect not only referred to clicking on the URL, but also to the proportion of log-ins to the program. Although a trend was observed about the effectiveness of adding a preview of new website content to a standard prompt, this only referred to persuading people to log in to the program. The findings of this study underline the importance of sending an email prompt relatively shortly after a first visit to the program. Furthermore, it is important to further focus on prompt content because the addition of a peripheral cue may add to the prompt’s effectiveness.

## Abbreviations

ANOVA: analysis of variance
CT: computer tailored
ELM: elaboration likelihood model
FFQ: Food Frequency Questionnaire
NP: no prompt
OR: odds ratio
QFV: Quantity-Frequency-Variability
RPHS: Regional Public Health Service
SP: standard prompt
SP+: standard+ prompt
SQUASH: Short Questionnaire to Assess Health-enhancing physical activity
URL: uniform resource locator
